# A few enlarged chloroplasts are less efficient in photosynthesis than a large population of small chloroplasts in *Arabidopsis thaliana*

**DOI:** 10.1038/s41598-017-06460-0

**Published:** 2017-07-18

**Authors:** Dongliang Xiong, Jianliang Huang, Shaobing Peng, Yong Li

**Affiliations:** 1 National Key Laboratory of Crop Genetic Improvement, MOA Key Laboratory of Crop Ecophysiology and Farming System in the Middle Reaches of the Yangtze River, College of Plant Science and Technology, Huazhong Agricultural University, Wuhan, Hubei 430070 China; 2 Research Group on Plant Biology under Mediterranean Conditions, Universitat de les Illes Balears, Carretera de Valldemossa Km 7.5, 07121 Palma de Mallorca, Illes Balears Spain; 3 Hubei Collaborative Innovation Center for Grain Industry, Yangtze University, Jingzhou, Hubei 434023 China

## Abstract

The photosynthetic, biochemical, and anatomical traits of *accumulation and replication of chloroplasts* (*arc*) mutants of *Arabidopsis thaliana* were investigated to study the effects of chloroplast size and number on photosynthesis. Chloroplasts were found to be significantly larger, and the chloroplast surface area exposed to intercellular air spaces (*S*_c_) significantly lower in the mutants than in their wild-types. The decreased *S*_c_ and increase cytoplasm thickness in the mutants resulted in a lower mesophyll conductance (*g*_m_) and a consequently lower chloroplast CO_2_ concentration (*C*_c_). There were no significant differences between the mutants and their wild-types in maximal carboxylation rate (*V*_cmax_), maximal electron transport (*J*_cmax_), and leaf soluble proteins. Leaf nitrogen (N) and Rubisco content were similar in both Wassilewskija (Ws) wild-type (Ws-WT) and the Ws mutant (*arc* 8), whereas they were slightly higher in Columbia (Col) wild-type (Col-WT) than the Col mutant (*arc* 12). The photosynthetic rate (*A*) and photosynthetic N use efficiency (PNUE) were significantly lower in the mutants than their wild-types. The mutants showed similar *A*/*C*_c_ responses as their wild-type counterparts, but *A* at given *C*_c_ was higher in Col and its mutant than in Ws and its mutant. From these results, we conclude that decreases in *g*_m_ and *C*_c_ are crucial to the reduction in *A* in *arc* mutants.

## Introduction

The chloroplast is one of the most important plant organelles and carries out many important functions such as fatty acid synthesis, nitrogen (N) and sulphur fixation, and especially photosynthetic carbon fixation^1^. During the process of leaf development, proplastids in meristematic cells first differentiate into primeval chloroplasts, then undergo subsequent divisions to produce a large population of small chloroplasts in mesophyll cells^2, 3^. Many genetic approaches to understand chloroplast division and development in mesophyll cells in the model plant, *Arabidopsis thaliana*, have been used in the past decades. It is widely believed that chloroplasts are derived from ancient cyanobacterial endosymbionts. Chloroplast size and the number of chloroplasts per cell are regulated by both genetic and environmental factors. Rapid advances have been made in research on the regulatory mechanisms of chloroplast division in recent years due, in part, to the isolation of *accumulation and replication of chloroplast* (*arc*) mutants in *A. thaliana* ^4–9^. *arc* mutants exhibit alterations in chloroplast size and number of mesophyll cells. In addition to genetic factors, the growth environment has been shown to play an important role in chloroplast size and number in mesophyll cells. Numerous studies have reported that chloroplast size and number increase under a high CO_2_ concentration^10–12^ but decrease under high temperature^13, 14^. In addition, several studies investigating the effects of enlarged chloroplasts on photosynthesis using *A. thaliana arc* mutants found that the photosynthetic rate (*A*) decreased in *arc* mutants^15^. Most recently, the decline of mesophyll conductance (*g*_m_) in *arc* mutants was observed by Weise, *et al*.^16^ However, the anatomical factors leading to a lower *g*_m_ in *arc* mutants are not clear yet.

Although the regeneration of ribulose-1,5-bisphosphate (RuBP) has been observed to limit *A* in several studies^17, 18^, *A* in well-grown C_3_ plants under light-saturated conditions is mainly considered to be limited by two factors under ambient CO_2_ concentrations: leaf biochemistry (*i.e*., the carboxylation capacity of ribulose-1,5-bisphosphate carboxylase/oxygenase; Rubisco) and/or CO_2_ supplementation^18^. In C_3_ plants, a large amount of total leaf N exists in chloroplasts, mainly forming photosynthetic proteins in the stroma. As a key enzyme in photosynthesis, Rubisco is exceptionally abundant, accounting for approximately half of total leaf N. Because of the large investment of leaf N in Rubisco and electron transport proteins (the latter with approximately 7% of total leaf N), a strong and positive correlation between *A* and leaf N content per leaf area is frequently observed^17^. Therefore, increasing the chloroplast volume per unit leaf area, and hence increasing the leaf N content, would potentially increase the rate of photosynthesis.

Under a given environmental condition (*i.e*. temperature and light), the carboxylation to oxygenation ratio of RuBP is determined by the CO_2_ concentration in the chloroplast (*C*_c_)^19–23^. CO_2_ molecules diffuse from the atmosphere into chloroplasts by overcoming a series of diffusion resistances, including the boundary layer, stomata and mesophyll resistances, which results in a remarkable drawdown in *C*_c_ compared to the atmospheric CO_2_ concentration. The diffusion conductances of stomata and mesophyll tissues are defined as stomatal conductance (*g*_s_) and *g*_m_, respectively. In the last 10–15 years, considerable efforts have been focused on the chloroplast features that determine g_m_. These have shown that there is a tight relationship between the area of chloroplast surface exposed to intercellular airspaces (*S*_c_) and *g*_m_^22, 24–26^. In mesophyll cells, chloroplasts are usually located next to the cytoplasmic membrane adjacent to intercellular air spaces, which was suggested to decrease resistance to CO_2_ diffusion^25^. Smaller chloroplasts are more flexible in movement than larger chloroplasts, especially under variable environmental conditions^27, 28^, which was suggested as the explanation why plants usually contain many small chloroplasts rather than a few large ones.

Although the important role of chloroplast number as well as chloroplast shape in *g*_m_ decreasing was suggested in the study of Weise *et al*.^16^, the reasons of low *A* and *g*_m_ in *arc* mutants are still unclear. As described above, both leaf structural properties, which were not quantified in their study, play the key role in determining *A*. In the present study, we used two *Arabidopsis arc* mutants and the corresponding wild-type plants to investigate the effects of chloroplast size and number on photosynthesis. Our objective was to evaluate whether a small number of enlarged chloroplasts is less beneficial to photosynthesis than a large population of small chloroplasts using leaf structural and biochemical analysis and 1-D mesophyll conductance model.

## Results

### Growth performance and photosynthetic characteristics

**Figure 1:**
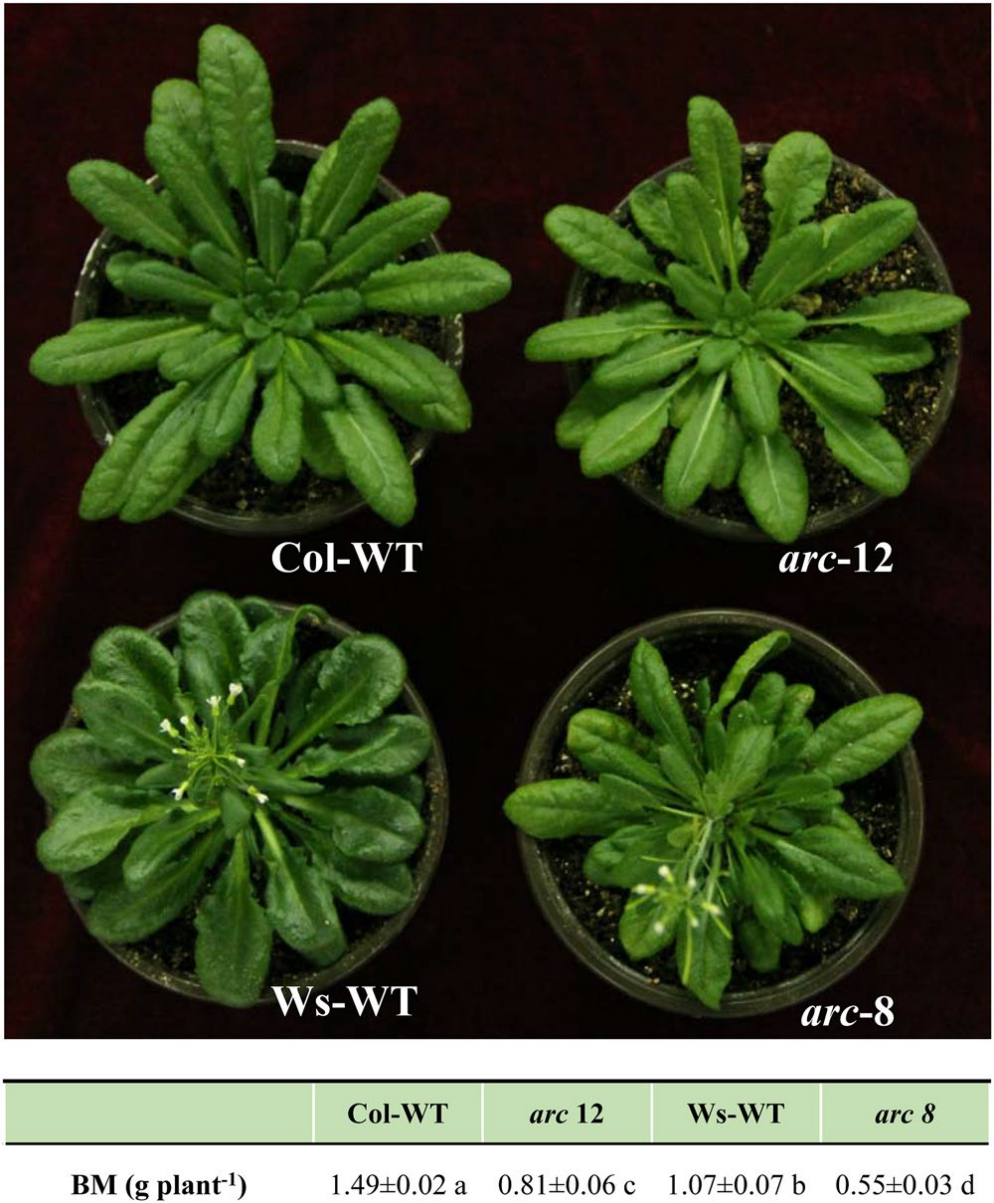
Phenotypes and biomass (BM) of plants 72 days after germination. The BM values shown are the mean ± SD of three replicates. The means were compared with a least significant difference (LSD) test; values followed by the same letter are not significantly different (*P* < 0.05).

To investigate the effects of chloroplast size and number on plant performance and photosynthesis, the photosynthetic characteristics of two *arc* mutants and their wild-types (Table 1) were analysed. Both mutants exhibited lower *A*, transpiration (*E*) and CO_2_ diffusion conductance than their wild-types, and consequently reduced biomass accumulation (Table 2 and Fig. 1). Compared with Columbia (Col) and Wassilewskija (Ws) wild-type plants, *g*_s_ was reduced by 42.4% and 61.0% in their respective mutants (*arc* 12 and *arc* 8) (Table 2). The intercellular CO_2_ concentration (*C*_i_) was similar in both *arc* 12 and Col-WT, although it was lower in *arc* 8 than in Ws-WT. Furthermore, *g*_m_ was determined using two independent methods and showed a good correlation in both (Table 2). Similar to *g*_s_, the *g*_m_ in the mutants was significantly lower than that in the wild-type plants, resulting in a greater drawdown of *C*_c_ from *C*_i_. Day respiration (*R*_d_) and CO_2_ compensation point in the absence of respiration (Γ*) were similar in the mutants and their wild-types.

**Table 1:** Details of the materials used in this study.

Symbol	Ecotype	Accessions	Chloroplast Number (/Cell)
Col-WT	Col-0	N60000	100
*arc* 12	Col-0	N16472	1–2
Ws-WT	Ws	N1601	83
*arc* 8	Ws	N284	45

The chloroplast numbers were obtained from the European Arabidopsis Stock Centre (NASC, http://arabidopsis.info/) with the accession number.

**Table 2:** Leaf functional characteristics.

	Col-WT	*arc* 12	Ws-WT	*arc* 8
*A* (μmol m^−2^ s^−1^)	7.69 ± 0.54 a	3.86 ± 0.40 c	4.72 ± 0.36 b	3.32 ± 0.23 c
*g*_t_ (mol m^−2^ s^−1^)	0.054 ± 0.012 a	0.015 ± 0.002 c	0.049 ± 0.001 b	0.015 ± 0.002 c
*g*_s_ (mol m^−2^ s^−1^)	0.085 ± 0.003 b	0.049 ± 0.008 c	0.100 ± 0.009 a	0.039 ± 0.012 c
*g*_m-Harley_ (mol m^−2^ s^−1^)	0.152 ± 0.041 a	0.022 ± 0.004 c	0.098 ± 0.010 b	0.028 ± 0.008 c
*g*_m-Ethier_ (mol m^−2^ s^−1^)	0.132 ± 0.016 a	0.021 ± 0.007 c	0.097 ± 0.008 b	0.029 ± 0.006 c
*g*_*m-anatomy*_ (mol m^−2^ s^−1^)	0.111 ± 0.003 a	0.069 ± 0.010 c	0.087 ± 0.005 b	0.065 ± 0.011 c
*C*_i_ (μmol mol^−1^)	292 ± 21 b	309 ± 17 b	341 ± 8 a	299 ± 29 b
*C*_c_ (μmol mol^−1^)	239 ± 27 b	132 ± 32 d	293 ± 7 a	172 ± 12 c
*E* (μmol m^−2^ s^−1^)	1.56 ± 0.21 a	0.92 ± 0.13 b	1.84 ± 0.24 a	0.96 ± 0.30 b
*R*_d_ (μmol m^−2^ s^−1^)	1.61 ± 0.16	1.54 ± 0.19	1.37 ± 0.11	1.26 ± 0.18
Г* (μmol mol^−1^)	41.3 ± 3.4	40.6 ± 2.7	39.5 ± 3.1	40.1 ± 3.3
*J* (μmol m^−2^ s^−1^)	56.0 ± 4.1 a	47.0 ± 8.5 b	33.7 ± 2.4 c	33.1 ± 1.4 c
*V*_cmax_ (μmol m^−2^ s^−1^)	30.5 ± 0.7 a	29.8 ± 3.0 a	18.1 ± 2.0 b	18.0 ± 0.9 b
*J*_cmax_ (μmol m^−2^ s^−1^)	57.8 ± 2.0 a	52.7 ± 4.2 a	35.3 ± 1.9 b	38.9 ± 0.5 b
PNUE (µmol g^−1^ N s^−1^)	9.18 ± 0.86 a	5.06 ± 0.39 c	7.82 ± 1.02 b	5.42 ± 0.47 c

The values shown are the mean ± SD of three replicates. The means were compared with a least significant difference (LSD) test; values followed by the same letter are not significantly different (*P* < 0.05). *A*, *g*_s_, *g*_m_, *g*_t_, *C*_i_ and *C*_c_ were measured at a CO_2_ concentration of 400 μmol mol^−1^ and a PPFD of 300 μmol m^−2^ s^−1^. *A*, photosynthetic rate; *g*_t_, total CO_2_ diffusion conductance; *g*_s_, stomatal conductance; *g*_m_, mesophyll conductance; *C*_i_, intercellular CO_2_ concentration; *C*_c_, chloroplast CO_2_ concentration; *E*, transpiration; *R*_d_, daytime mitochondrial respiration rate; Г*, CO_2_ photo-compensation point; *J*, electron transport rate; *V*_cmax_, maximum velocity of carboxylation; *J*_cmax_, maximum electron transport; PNUE, photosynthetic N use efficiency.

**Figure 2:**
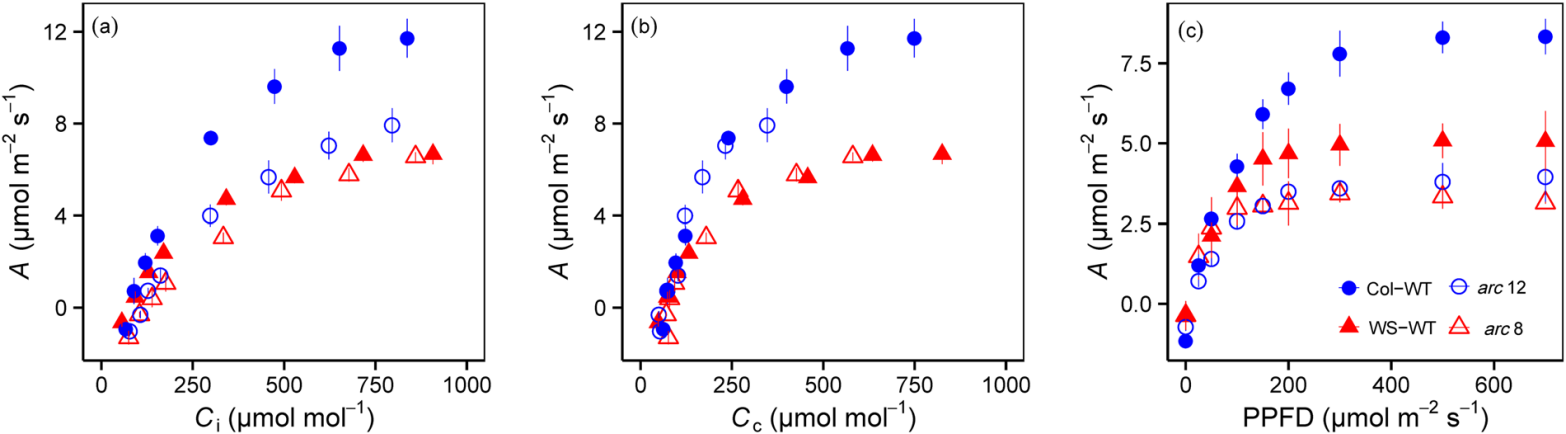
*A*/C_i_ (**a**), *A*/C_c_ (**b**) and light (**c**) response curves of the mutants and their wild-types. The values shown are the mean ± SD of three replicates.

*A* in the mutants was generally lower than that in their wild-types across the supplied CO_2_ concentrations (Fig. 2a). Interestingly, the maximal *A* from the *A*/*C*_i_ curves (*A*_max_) in *arc* 8 was comparable with that in Ws-WT, although *A*_max_ was lower in *arc* 12 than in Col-WT. The mutants and their respective wild-types generally showed similar *A*/C_c_ response curves; Col-WT and arc12 showed a higher *A* than Ws-WT and arc8 at a given C_c_ (Fig. 2b). The maximum velocity of carboxylation (*V*_cmax_) and maximum electron transport (*J*_cmax_), calculated from the *A*/*C*_i_ curves, were similar in the mutants and their wild-types (Table 2). Moreover, the light-saturated *A* and light saturation point determined from the light response curves were significantly lower in the mutants and their wild-types (Fig. 2c).

**Figure 3:**
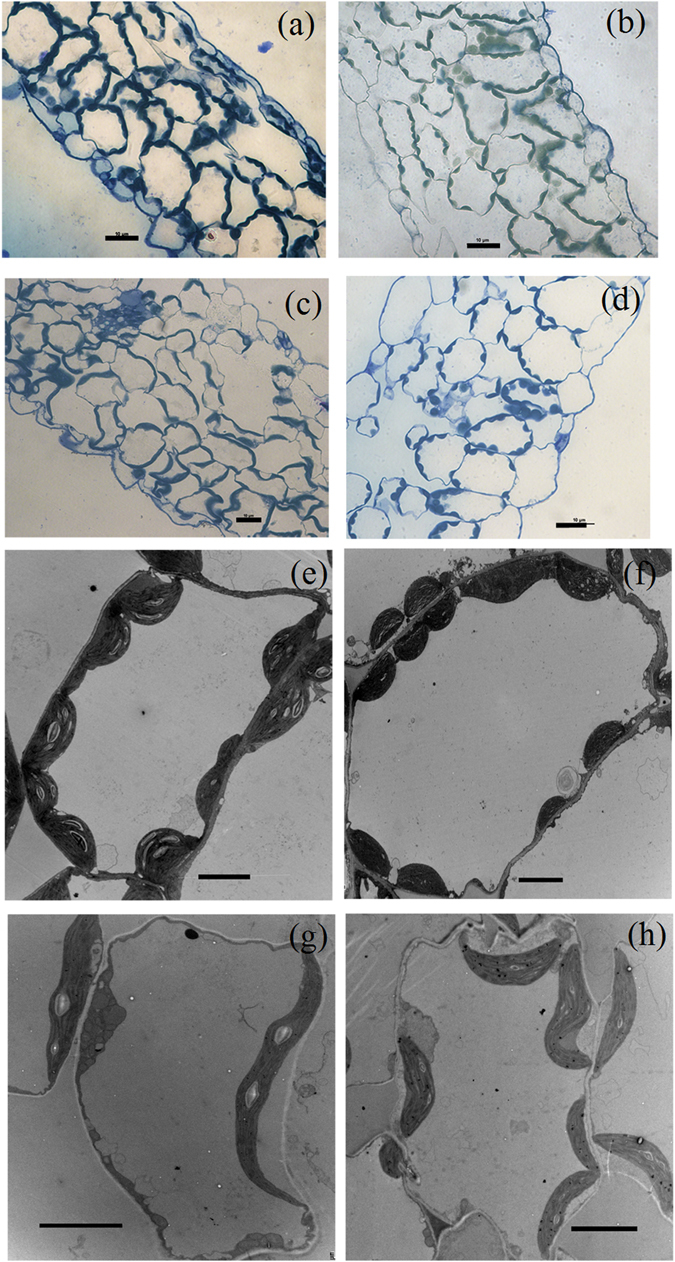
Light (**a**–**d**) and transmission electron (**e**–**h**) microscope images of Col-WT (**a**,**e**), Ws-WT (**b**,**f**), *arc*12 (**c**,**g**) and *arc* 8 (**d**,**h**) leaves. Bars represent 10 μm in (**a**–**d**) and 5 μm in (**e** and **h**).

**Figure 4:**
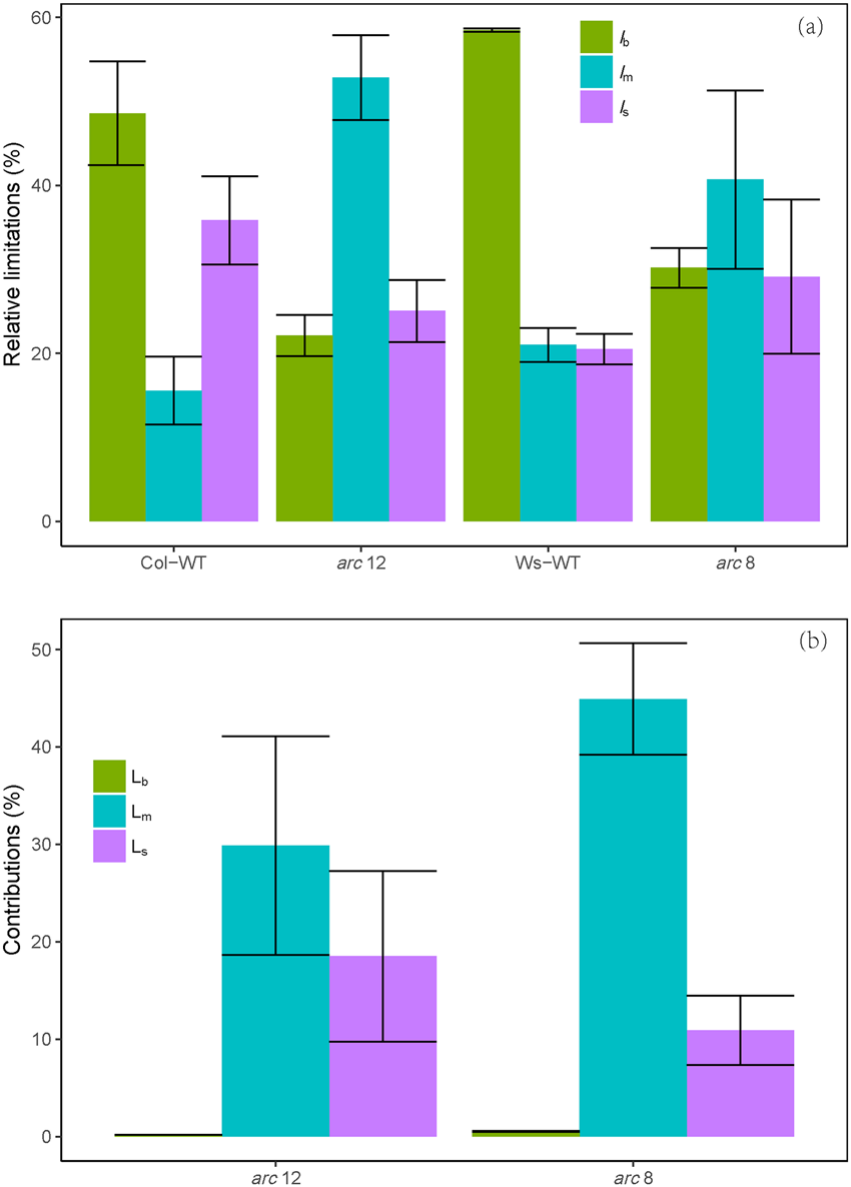
(**a**) quantitative relative limitations of stomatal conductance (*l*_s_), mesophyll conductance (*l*_m_) and biochemical factors (*l*_*b*_) and (**b**) the contributions of stomatal conductance (L_s_), mesophyll conductance (L_m_) and biochemical factors (L_b_) to relative changes in light-saturated photosynthetic rate (*A*) in mutants (d*A*/*A* = (*A*_wild-type_ − *A*_*arc*_)/*A*_wild-type_). Where the *A*_wild-type_ and *A*_*arc*_ are the *A* in wild-types and mutants, respectively.

Differences in plant growth and photosynthetic parameters were also observed between accessions. The biomass, *A*, *g*_t_, *g*_m_, *V*_cmax_, *J*_cmax_, *A*_max_ and electron transport rate (*J*) of Col-WT were higher than those of Ws-WT. Conversely, *g*_s_, *C*_i_ and *C*_c_ were lower in Col-WT than in Ws-WT (Table 2, Figs 1 and 3a). The quantitative limitation analysis (Fig. 4) showed that the decreases of *A* in two mutants were mostly due to a mesophyll conductance limitation (*L*_m_, 29.9% in *arc* 12; and 49.8% in *arc* 8), followed by a stomatal conductance limitation (*L*_s_, 18.5% in *arc* 12; and 10.9% in *arc* 8), while the biochemical limitation (*L*_b_, 0.17% in *arc* 12; and 0.51% in *arc* 8) was of minor importance in both mutants.

### Leaf N content, chlorophyll content, and Rubisco content

The leaf N and Rubisco content per leaf area were significantly higher in Col-WT than in *arc* 12, but there were no significant differences between Ws-WT and *arc* 8 (Table 3). There was no difference in chlorophyll content per leaf area, chlorophyll a/b, and soluble protein content per leaf area between the mutants and their wild-types. All these chemical traits, except for soluble proteins, were significantly higher in Col than in Ws.

**Table 3:** Leaf chemical features

	Col-WT	*arc* 12	Ws-WT	*arc*8
Leaf N content (g m^−2^)	0.847 ± 0.021 a	0.762 ± 0.047 b	0.604 ± 0.036 c	0.613 ± 0.006 c
Chl a + b (g m^−2^)	0.239 ± 0.004 a	0.233 ± 0.016 a	0.213 ± 0.033 b	0.225 ± 0.008 b
Chl a/b	2.09 ± 0.11 a	2.04 ± 0.05 a	1.57 ± 0.05 b	1.63 ± 0.14 b
Rubisco (g m^−2^)	0.510 ± 0.027 a	0.446 ± 0.052 b	0.244 ± 0.038 c	0.212 ± 0.022 c
Protein (g m^−2^)	0.80 ± 0.02	0.80 ± 0.03	0.76 ± 0.04	0.74 ± 0.11

The values shown are the mean ± SD of three replicates. The means were compared with a least significant difference (LSD) test; values followed by the same letter are not significantly different (*P* < 0.05).

### Leaf anatomical and structural features

There were no significant differences between the mutants and their wild-types in leaf thickness (*T*_leaf_), mesophyll thickness (*T*_mes_), cell wall thickness (*T*_cell-wall_), the mesophyll surface area exposed to intercellular air spaces per leaf area (*S*_m_), chloroplast planar area per planar cell area (P_chl_), chloroplast stroma thickness (*T*_str_) or mesophyll tissue occupied by the intercellular air spaces (*f*_ias_) (Table 4). As expected, chloroplast size (cross-sectional area from microscopy images) was greater in the mutants than in their wild-types, and *S*_c_ was lower in the mutants than in their wild-types. The cytoplasm thickness (*T*_cyt_) showed a significantly larger in mutants than in wide types. The leaf mass per area (LMA) was similar in both Col-WT and *arc* 12, but greater in Ws-WT than in *arc* 8.

**Table 4:** Leaf anatomical characteristics.

	Col-WT	*arc* 12	Ws-WT	*arc* 8
LMA (g m^−2^)	11.38 ± 0.73 b	11.85 ± 0.26 b	14.47 ± 0.49 a	12.93 ± 0.80 b
*T*_leaf_ (μm)	80.1 ± 5.6	79.6 ± 11.3	81.1 ± 7.2	75.9 ± 4.7
*T*_mes_ (μm)	68.4 ± 3.4	67.8 ± 6.3	71.3 ± 7.6	67.6 ± 5.1
*T*_cell wall_ (μm)	0.174 ± 0.006 b	0.181 ± 0.004 b	0.193 ± 0.002 a	0.199 ± 0.007 a
*S*_m_ (m^2^ m^−2^)	9.02 ± 0.77 a	8.52 ± 0.60 a	7.77 ± 0.54 b	7.38 ± 0.61 b
*S*_c_ (m^2^ m^−2^)	8.17 ± 0.54 a	5.26 ± 0.60 c	6.31 ± 0.39 b	5.48 ± 0.62 c
*T*_cyt_ (μm)	0.100 ± 0.003 b	0.121 ± 0.008 a	0.097 ± 0.009 b	0.123 ± 0.007 a
*T*_str_ (μm)	1.98 ± 0.31	2.13 ± 0.40	1.81 ± 0.31	2.22 ± 0.22
Chloroplast size (μm^2^)	14.6 ± 2.1 c	198.2 ± 4.7 a	15.1 ± 2.3 c	34.4 ± 3.3 b
P_chl_ (m^2^ m^−2^ mesophyll)	44.3 ± 5.1 a	41.9 ± 2.8 a	31.8 ± 2.9 b	30.6 ± 4.5 b
*f*_ias_ (%)	23.4 ± 3.4	19.9 ± 2.9	22.7 ± 1.7	24.6 ± 3.3

The values shown are the mean ± SD of three replicates. The means were compared with a least significant difference (LSD) test; values followed by the same letter are not significantly different (*P* < 0.05). LMA, leaf mass per leaf area; *T*_leaf_, leaf thickness; *T*_mes_, mesophyll thickness; *T*_cell wall_, _cell wall_ thickness; *S*_m_, mesophyll cell surface area face to intercellular air space per leaf area; *S*_c_, chloroplast surface area face to intercellular air space per leaf area; *T*_cyt_, cytoplasm thickness; *T*_str_, chloroplast stroma thickness; *P*_chl_, chloroplast planar area per planar cell area; *f*_ias_, mesophyll tissue occupied by the intercellular air spaces.

There were no significant differences in *T*_leaf_, *T*_mes_, chloroplast size, or *f*_ias_ between Col-WT and Ws-WT. LMA and *T*_cell-wall_ were both greater in *arc* 8 than in Col-WT, whereas *S*_m_, *S*_c_, and chloroplast planar area per planar cell area (P_chl_) were lower in Ws-WT.

### Limitation of leaf anatomical traits to mesophyll conductance

**Figure 5:**
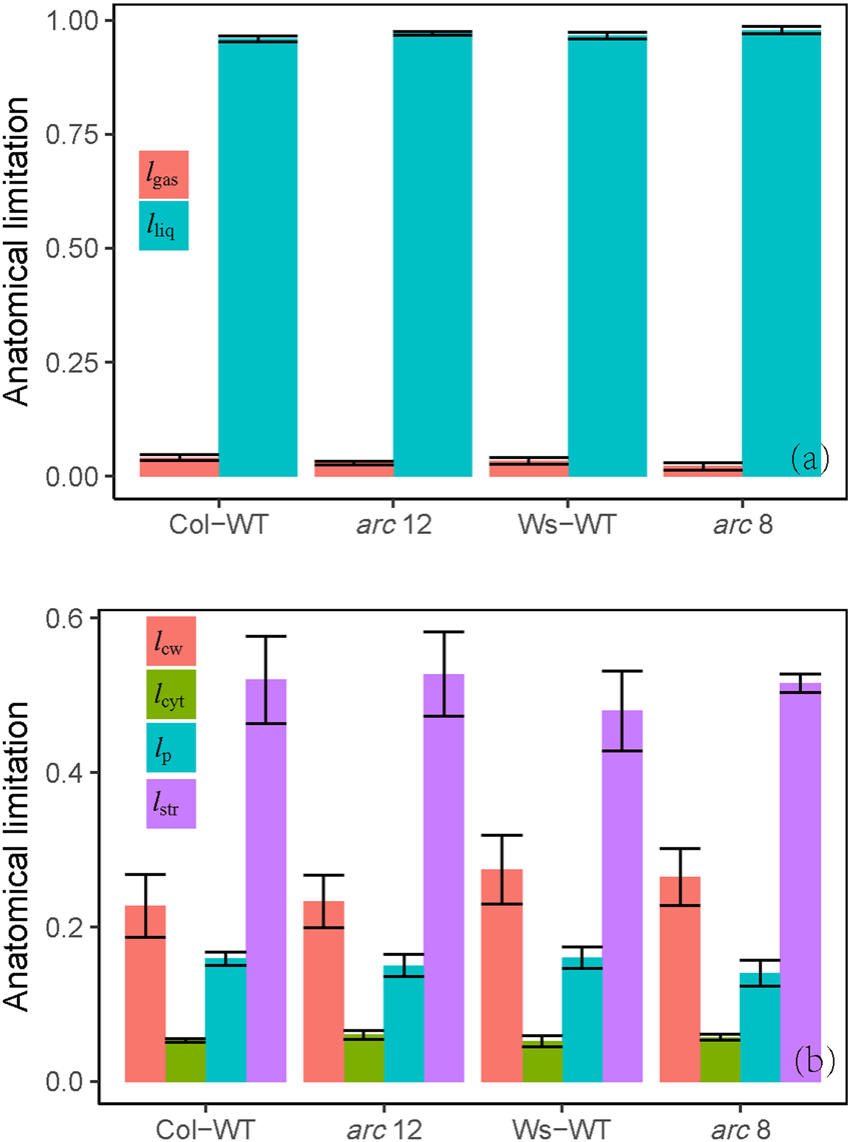
Limitation of mesophyll conductance due to anatomical constraints. (**a**) Share of the overall *g*_m_ limitation by gas (*l*_gas_) and liquid phase (*l*_liq_) and (**b**) the liquid-phase limitation among its components: cell wall (*l*_cw_), cytosol (*l*_cyt_), plasmalemma and chloroplast envelope membranes (*l*_p_), and chloroplast stroma (*l*_str_). The *l*_ias_ was calculated as *g*_m_/*g*_ias_ and the liquid-phase limitations of each components were calculated as *l*_i_ = *g*_m_/(*g*_i_ **·** *S*_c_).

The *g*_m_ values calculated from anatomical traits agreed well with the values estimated from whether Harley method or Ethier method (Table 2). From the different components of the whole CO_2_ diffusion pathway, the limitations of *g*_m_ were calculated (Fig. 5a,b). Intercellular air spaces (*f*_IAS_) represented less than 5.0% of CO_2_ diffusion resistance (max. 4.1% in Col-WT and min. 2.2% in *arc* 8). In the cellular phase, the stroma represented about half of diffusion resistance to CO_2_ (range from 48% in Ws-WT to 53% in *arc* 12). Otherwise, cell wall and membrane (including Plasmalemma and chloroplast envelope) accounted for ~40% of limitations. The individual components diffusion resistances were quite stable among genotypes.

## Discussion

In C_3_ plants, under ambient temperature (25 °C) and CO_2_ concentration (380 ppm), light-saturated photosynthesis is primarily limited by Rubisco carboxylation capacity, CO_2_ diffusion conductance and the RuBP regeneration rate from ambient to chloroplasts^17^. In the present study, we found that chloroplast size and number affect *A* by changing CO_2_ diffusion conductance.

### Variation in biochemical features and their effects on photosynthesis

Tight correlations between *A* and leaf N content and Rubisco content per leaf area were frequently observed in previous studies^29^. Although the leaf N and Rubisco contents slightly decreased in *arc*12 and did not decrease in *arc*8, the *A* in both mutants was dramatically lower than that in their wild-types. Our results indicate that the decrease in *A* in the mutants is not, at least not mainly, caused by the changes in leaf N and/or Rubisco content. However, the relatively higher leaf N, Rubisco and chlorophyll content per leaf area in Col-WT compared to Ws-WT at least partly accounted for the higher *A* in Col-WT (Table 2). Interestingly, in the current study leaf N content, Rubisco and chlorophyll content per leaf area were related to P_chl_, and the higher Rubisco and chlorophyll content in Col were related to their larger chloroplast volumes per unit leaf (represented as P_chl_). Our results suggest that Rubsico and chlorophyll concentrations in chloroplasts may tend to be conservable, which has also been mentioned in previous studies^24, 30^. Otherwise, the larger total chloroplast volume in Col-WT than in Ws-WT is mainly due to the smaller size of the mesophyll cells and a greater number of chloroplasts in Col-WT (Fig. 3 and Table 4). It can be observed from Fig. 3 that a smaller proportion of mesophyll cell surface was covered with chloroplasts, which was the major reason for the lower *S*_c_ in the mutants.

### Variation in CO_2_ diffusion conductance

Compared with the wild-type plants, a lower *g*_s_ was observed in both mutants. The decreased *g*_s_ in mutants may be due to changes in stomatal size, density, or opening status, with opening status usually being regulated by leaf water status (i.e leaf water potential). However, in the current study, we did not estimate stomatal features or opening status.

**Figure 6:**
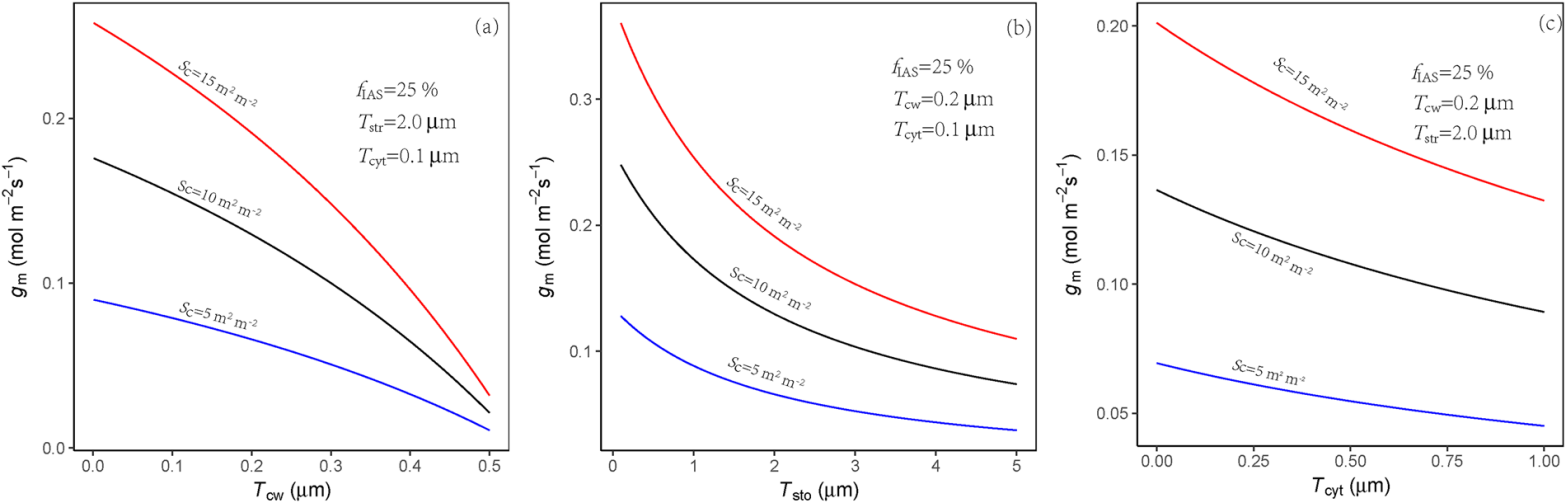
Modelled mesophyll conductance at 25 °C response to cell wall thickness (*T*_cw_), chloroplast stroma thickness (*T*_str_) and cytoplasm thickness (*T*_cyt_). Membrane conductance was the same in all simulations. *f*_ias_, volume fraction of intercellular air space and *S*_c_, area of chloroplast surface exposed to intercellular airspace.

*g*_m_, which was calculated using three independent methods, was found to correlate well with *A*. Photosynthetic limitation analysis (Fig. 4) revealed that the decrease of mesophyll conductance is the most important factor limiting *A* in mutants. Mesophyll structural features, including *T*_cell-wall_, *S*_m_, and *S*_c_ are believed to play a central role in determining *g*_m_^22, 24, 26, 31^. Using the 1-D anatomical model, we analyzed the impacts of leaf anatomical traits on mesophyll conductance by considering all major leaf anatomical traits as described by Tosens *et al*.^32^ and Tomas *et al*.^26^. We note that many previous studies demonstrated that cell wall porosity and aquaporins can dramatically influence *g*_m_, however, the 1-D anatomical model was failed to estimate their contributions. Although the absolute values were not exactly the same, the variable pattern of measured and modeled *g*_m_ among estimated genotypes were quite similar (Table 2), which suggests that reduction of *g*_m_ in mutants is mainly related to leaf anatomical traits. The partial limitation analysis (Fig. 5) of *g*_m_ showed that *T*_str_, *T*_cw_, and biological membranes are three most important factors limiting *g*_m_. However, there were no significant difference of those traits among wild types and mutants. By modeling the influences of *T*_str_, *T*_cyt_ and *T*_cw_ on modeling *g*_m_ with a variable *S*_c_ (Fig. 6), we found that *S*_c_ can strongly influence *g*_m_. A slight decrease of *S*_c_ leads a significant reduce in *g*_m_ in leaves with a relative thin cell wall (i.e. less than 0.2 µm) or chloroplast stroma (i.e. less than 2 µm) like the *A. thaliana* leaves (Table 4) estimated here. The *S*_c_ of the mutants was significantly lower than that in their wild-types, which resulted in decreased *g*_m_ in the mutants. Otherwise, the distance between cell membrane and chloroplasts (*T*_cyt_) was increased significantly in the mutants (Table 4 and Fig. 3), which also potentially increased the CO_2_ diffusion pathway and then decreased the *g*_m_ (Fig. 6c). Therefore, our results highlight the significant effects of chloroplast size and number on *T*_cyt_ and *S*_c_ and, consequently, *g*_m_ and *A*.

### Differences in *A*/*C*_i_ and *A*/*C*_c_ curves

*A*/*C*_i_ curves are frequently used to analyse photosynthetic limitations, including Rubisco carboxylation capacity at low *C*_i_ values and RuBP regeneration rate as well as the utilization of photosynthates at high *C*_i_ values^33^. In the present study, the *A*/*C*_i_ curves of the mutants and their wild-types were different, such that the mutants had reduced *A* compared to their wild-types (Fig. 2a). *A* was generally not significantly improved in the two wild-types when C_i_ > 600 μmol mol^−1^; in contrast, it was significantly higher in the two mutants. This suggested that the CO_2_ saturation points in the mutants were higher than those in their wild-types. In *arc* 12, *A* gradually increased across the supplied CO_2_ concentrations, and CO_2_ was not saturated at its highest *C*_i_ value of approximately 800 μmol mol^−1^.

As suggested earlier, restricted CO_2_ diffusion conductance accounted for the low *A* in the mutants. If this is the case, the *A*/C_c_ curves would be similar in the mutants and their wild-types, which was indeed observed in the present study (Fig. 2b). Moreover, *A* in the mutants gradually increased with increasing CO_2_ concentrations and reached 6.55 μmol m^−2^ s^−1^ in *arc* 8 which was similar to the 6.66 μmol m^−2^ s^−1^ in its wild-type (Fig. 2a). Because CO_2_ was not saturated for *arc* 12, *A*_max_ in *arc* 12 (7.56 μmol m^−2^ s^−1^) was lower than that in Col-WT (11.72 μmol m^−2^ s^−1^). Therefore, the results illustrated by the *A*/*C*_i_ and *A*/C_c_ curves also demonstrate that chloroplast size and number can significantly affect *A* through by CO_2_ diffusion conductance.

### Implications

The question of why photosynthetic mesophyll cells in higher plants contain numerous small chloroplasts rather than one or a few larger ones, has been asked and pursued by many researchers^27, 34^. It has been suggested that small chloroplasts can rapidly change their positions or directions in response to changing irradiance in order to maximize their utilization of limiting irradiance or to minimize photodamage under excess light conditions^27^. Our study findings highlight the fact that a large population of small chloroplasts in mesophyll cells can benefit CO_2_ diffusion conductance and the consequent *A*. Moreover, the photosynthetic N use efficiency (PNUE) was also significantly higher in plants with a large population of small chloroplasts. This suggests that increasing chloroplast number and decreasing chloroplast size would be a potential approach to improve N use efficiency in plants, especially in crops.

Numerous studies have reported that PNUE decreases with increasing leaf N content per leaf area, and much effort has focused on exploring the underlying mechanisms. Lower Rubisco activation status and insufficient CO_2_ supplementation are frequently observed under high N conditions. It was reported that chloroplast size is significantly increased by high N supplementation in many species^22, 35^. Moreover, constant Rubisco and chlorophyll concentration in chloroplasts were observed in the present study and by Li, *et al*.^21^. Thus, large chloroplasts will have a smaller surface area to volume ratio, leading to a reduction in exposure to intercellular airspaces and thus a lower rate of CO_2_ diffusion into the chloroplast.

## Conclusion

The decreased *A* in *arc* mutants was due to their lower *C*_c_. The decreased *C*_c_ in the mutants was related to reduced *g*_m_, which was strongly constrained by the lowered *S*_c_. From these results, we conclude that the decrease in *g*_m_ was crucial for the decrease in *C*_c_ and *A* in *arc* mutants.

## Materials and Methods

### Plant materials

*A. thaliana* L. (Heynh) mutants N16472 (*arc 12*) and N284 (*arc 8*), and their background lines N60000 (Col-08) and N1601 (Ws-2), respectively, were obtained from the European Arabidopsis Stock Centre (NASC, http://arabidopsis.info/) (Table 1). Seeds were incubated at 4 °C for 2 days, then sown in pots filled with a substrate containing peat, perlite and vermiculite (2:1:1 v/v)^36^. Pots with plastic trays for sub-irrigation were placed in a growth chamber under controlled condition (8:16 h photoperiod, 23:19 °C day: night temperature, light at 350 ± 47 μmol m^−2^ s^−1^ and relative humidity at 78 ± 13%). The plants were watered when needed. From 4 weeks after germination, 50 ml of half-strength Hoagland solution^37^ was added to each pot once per week. Nine weeks after germination, the plants were used for subsequent measurements.

### Gas exchange and chlorophyll fluorescence measurements

A portable photosynthesis system equipped with an integrated fluorescence chamber (LI-6400XT; LI-COR Inc., Lincoln, NE, USA) was used to obtain simultaneous measurements of leaf gas exchange and chlorophyll fluorescence. To minimize the effects of leaf position and leaf age, measurements were taken from newly and fully expanded leaves. Photosynthesis was initiated at a leaf temperature of 23 °C, a leaf-to-air vapour pressure deficit (VPD) of 1.1 ± 0.3 kPa, a photosynthetic photo flux density (PPFD) of 300 μmol m^−2^ s^−1^ with 10% blue light, and a CO_2_ concentration of 400 μmol mol^−1^ with a CO_2_ mixture. After equilibration to a steady state, gas exchange parameters, steady state fluorescence (*F*_s_) and maximal fluorescence (*F*_m_’) were recorded with a light-saturating pulse of 8000 μmol m^−2^ s^−1^. The actual photochemical efficiency of photosynthetic system II (Φ_PSII_) was calculated as:

1 $${{\rm{\Phi }}}_{{\rm{PSII}}}=({F}_{{\rm{m}}}\mbox{'}-{F}_{{\rm{s}}})/{F}_{{\rm{m}}}\mbox{'}$$

The *J* was calculated as:

2 $$J={{\rm{\Phi }}}_{{\rm{PSII}}}\times {\rm{PPFD}}\times {\rm{\alpha }}\times {\rm{\beta }}$$

where α is the leaf absorption and β is the partition ratio of absorbed quanta between photosystems I and II. The product of α and β was determined from the slope of the linear correlation between the quantum efficiency of gross CO_2_ uptake (Φ_CO2_) and 1/4Φ_PSII_, which was obtained by simultaneously measuring leaf gas exchange and chlorophyll fluorescence at varying light intensities under nonphotorespiratory conditions (<2% O_2_). Eight dead leaves were used to estimate the leakage effects of the chamber as described in our previous study^22^.

The variable *J* method^37^ was used to calculate *C*_c_ and *g*_m_:

3 $${{\rm{C}}}_{{\rm{c}}}=\frac{{{\rm{\Gamma }}}^{\ast }(J+8(A+{R}_{{\rm{d}}}))}{J-4(A+{R}_{{\rm{d}}})}$$

4 $${g}_{{\rm{m}}}=\frac{A}{{C}_{{\rm{i}}}-{C}_{{\rm{c}}}}$$

In this study, the Laisk method was used to estimate Γ* and *R*_d_. Briefly, the *A*/*C*_i_ curves were measured under three light conditions (50, 100 and 200 μmol m^−2^ s^−1^)^38^. The coordinates of the intersection point of three *A*/*C*_i_ curves were considered to be *C*_i_^*^ (the apparent CO_2_ photocompensation point; *x*-axis) and *R*_d_ (*y*-axis), and Γ* was calculated as:

5 $${{\rm{\Gamma }}}^{\ast }={C}_{{\rm{i}}}^{\ast }+\frac{{R}_{{\rm{d}}}}{{g}_{{\rm{m}}}}$$

In this study, the light response curves and CO_2_ response curves under ambient O_2_ condition were also measured. The CO_2_ concentration for the light response curves was set as 400 μmol mol^−1^, and the PPFDs were set across a series of 700, 500, 300, 200, 150, 100, 50 and 0 μmol m^−2^ s^−1^. The light conditions for the CO_2_ response curves were set as 300 μmol m^−2^ s^−1^ with 10% blue light, and the CO_2_ concentrations in the reference chamber were set across a series of 400, 200, 150, 100, 50, 400, 600, 800 and 1000 μmol mol^−1^. Calculation of *g*_m_ was also conducted using the method of Ethier & Livingston (2004) by using the CO_2_ response curves, which rely on the gas exchange measurements, adjusting the Farquhar model^17^ to extract, in conjunction with *V*_c,max_ and the *g*_m_. In the current study, the total CO_2_ diffusion conductance was calculated as: *g*_t_ = 1/(1/*g*_s_ + 1/*g*_m_), and the *g*_m_ values from the variable *J* method were used.

### Chlorophyll and leaf N content

For the chlorophyll content, leaf tissues were harvested using a circular punch that yields 0.5 cm-diameter leaf discs. Then, chlorophyll was extracted from the leaf discs using 95% (v/v) ethanol (analytically pure, Sinopharm Chemical Reagent Co., Ltd), and the extracted chlorophyll concentration was measured using a spectrophotometer (UV2102, Unico, Shanghai, China)^23^. For measurements of leaf N content, following photo-scanning, leaves were oven dried at 80 °C to a constant weight. The dried samples were digested with by the micro-Kjeldahl method, and then the N concentrations were measured using a discrete wet chemistry analyser (SmartChem® 200, AMS-Westco, Rome, Italy). The leaf area was determined using Image-J software (Wayne Rasband/NIH, Bethesda, MD, USA), and the leaf mass per area (LMA) was calculated as the ratio of leaf dry mass to leaf area.

### Rubisco content

The Rubisco concentration was measured using the sodium dodecyl sulphate polyacrylamide gel electrophoresis (SDS–PAGE) method^22, 23^. Leaf tissue was harvested using a circular punch, and immersed in liquid N. Samples were ground in liquid N and homogenized with an extraction buffer containing 50 mM Tris-HCl (pH 8.0), 5 mM β-mercaptoethanol, and 12.5% glycerol (v/v). After centrifugation at 1500 g for 15 min at 4 °C, the supernatants were mixed with an extraction buffer containing 2.0% (w/v) SDS solution, 4% (v/v) β-mercaptoethanol, and 10% (v/v) glycerol. Then, the solution was immediately boiled in a water bath for 1 min. The samples were loaded onto an SDS-PAGE gel containing a 4% (w/v) stacking gel, and a 12.5% (w/v) separating gel. After electrophoresis (DYY-11, Beijing Liuyi Instrument Factory, Beijing, China), the gels were washed several times with deionized water before being stained in 0.25% Coomassie blue staining solution for 9 h and then destained until the background was colourless. Both the large and small subunits were transferred into a 10 ml cuvette containing 2 ml of formamide and incubated in a 50 °C water bath for 8 h. The absorbance of the washed solution was measured at 595 nm (Infinite M200, Tecan U.S., Inc., Männedorf, Switzerland) using the background gel as a blank and bovine serum albumin (BSA) as a protein standard.

### Microscopy analysis

After gas exchange measurements, five small leaf discs approximately 1.2 × 4.0 mm were immediately removed from the leaf section inside the chamber with a razor blade, taking care to avoid the midveins. The leaf discs were infiltrated in a vacuum chamber (DZF-6050, Shanghai Hasuc Co. Ltd, Shanghai, China) with the fixative 2.5% glutaric aldehyde in 0.1 M phosphate buffer (pH = 7.6) at 4 °C, and then the samples were stored at 4 °C until analysis. Samples were cut using a fully automated rotary microtome (Leica RM2265, Leica Microsystems, Milton Keynes, UK) and were examined at 100 × magnification with an Olympus IX71 light microscope (Olympus Optical, Tokyo, Japan) after staining with 1% (w/v) toluidine blue O in 1% (w/v) Na_2_B_4_O_7_. Transmission images were obtained using a transmission electron microscope, H-7650 (Hitachi-Science and Technology, Tokyo, Japan). For both light and electron microscopes, three plants for each genotype were measured.

As described by Evans *et al*.^39^, the total cross-sectional area of mesophyll tissues (*S*_mes_) and intercellular air space area (*S*_ias_) and the width of the analysed leaf cross section (*L*) in light microscope images, the total length of the mesophyll cell wall exposed to the intercellular air space (*l*_m_) in light and electron microscope images, the total length of the chloroplasts touching the plasma membrane appressed to the intercellular air space (*l*_c_), and the thickness of cell wall (*T*_cw_), cytoplasm (*T*_cyt_) and chloroplast stroma (*T*_str_) in electron microscopes were measured using Image J software (National Institute of Health, Bethesda, MD, USA). The volume fraction of intercellular air space (*f*_ias_) was calculated as:

6 $${f}_{{\rm{ias}}}=\frac{{{\rm{S}}}_{{\rm{ias}}}}{{{\rm{S}}}_{{\rm{mes}}}}$$

*S*_m_ and *S*_c_ were then calculated as follows:

7 $$S=\frac{l}{L}\times F$$

where *S* is *S*_m_ or *S*_c_, *l* is *l*_m_ or *l*_c_, and *F* is the curvature correction factor. To convert the length in cross-sections to the surface area, *F* was measured and calculated for each genotype, as described by Thain^39^ for spongy and palisade cells. The curvature correction factor ranged from 0.95 to 1.04 for spongy cells and from 1.18 to 1.35 for palisade cells. There was no significant difference between the correction factors obtained for *S*_c_ and *S*_m_^40^.

### mesophyll conductance modeled from anatomical characteristics

Since *g*_m_ is affected by leaf anatomical traits, models have been developed that relied on anatomical and physical parameters. In those models, typically, *g*_m_ was estimated by dividing diffusivity of each individual component along the diffusion path^26, 32^. First, *g*_m_ is divided in a gas-phase conductance between the sub-stomatal cavities and the outer surface of cell walls (*g*_ias_), and a liquid-phase conductance between the outer surface of the cell walls and the site of carboxylation in the chloroplast stroma (*g*_liq_):

8 $${{\rm{g}}}_{{\rm{m}}}=\frac{1}{\frac{1}{{g}_{{\rm{ias}}}}+\frac{R{T}_{{\rm{k}}}}{H\cdot {g}_{{\rm{liq}}}}}$$

where *R* is the gas constant, *T*_k_ is the absolute temperature, and *H* is the Henry constant.

The *g*_ias_ is calculated based on the *f*_ias_ and the diffusion path length in the gas phase (∆*L*_ias_), which is assumed to be half of the mesophyll thickness:

9 $${g}_{{\rm{ias}}}=\frac{{D}_{{\rm{a}}}\cdot {f}_{{\rm{ias}}}}{{\rm{\Delta }}{L}_{{\rm{ias}}}\cdot {\rm{\varsigma }}}$$

where *D*_a_ (m^2^ s^−1^) is the diffusion coefficient for CO_2_ in the gas phase (1.51 × 10^−5^ at 25 °C), and ς is the diffusion path tortuosity (mm^−1^), which was fixed at 1.57 as in previous studies^26, 32^.

The *g*_liq_ is calculated as the sum of serial diffusion resistance (*r*):

10 $${g}_{{\rm{liq}}}=\frac{1}{\sum \frac{1}{{g}_{{\rm{i}}}}}\cdot {S}_{{\rm{c}}}$$

where *g*_i_ is the conductance of cell wall, plasmalemma, cytosol, chloroplast envelope, or chloroplast stroma. The conductance of a given component of the diffusion pathway can be calculated as:

11 $${g}_{{\rm{i}}}=\frac{{D}_{{\rm{w}}}\cdot {p}_{{\rm{i}}}\cdot {\gamma }_{{\rm{i}}}}{{\rm{\Delta }}{L}_{{\rm{i}}}}$$

where *D*_w_ is the aqueous phase diffusion coefficient for CO_2_, *p*_i_ is the effective porosity which variable with cell wall thickness, ∆*L*_i_ is the diffusion path length that is usually represented by the thickness of a component, and γ_i_ is a dimensionless factor accounting for a decrease of diffusion conductance in the cytosol and in the stoma compared with free diffusion in water. Because the structural parameters of plasma membrane and chloroplast envelope are impossible to estimate from light or electron microscopes images, an estimated of 0.0035 m s^−1^ for both plasma membrane conductance (*g*_pl_) and chloroplast envelope conductance (*g*_en_) were used as previous studies^26^.

### Quantitative limitation analysis of photosynthesis

Relative photosynthetic limitations including stomatal (*l*_s_), mesophyll (*l*_s_) and biochemical (*l*_b_) relative limitations were calculated according to Grassi and Magnani^41^.

12 $${l}_{{\rm{s}}}=\frac{{g}_{{\rm{t}}}/{g}_{{\rm{s}}}\cdot \partial A/\partial {C}_{{\rm{c}}}}{{g}_{{\rm{t}}}+\partial A/\partial {C}_{{\rm{c}}}}$$

13 $${l}_{{\rm{m}}}=\frac{{g}_{{\rm{t}}}/{g}_{m}\cdot \partial A/\partial {C}_{c}}{{g}_{{\rm{t}}}+\partial A/\partial {C}_{{\rm{c}}}}$$

14 $${l}_{{\rm{b}}}=\frac{{g}_{{\rm{t}}}}{{g}_{{\rm{t}}}+\partial A/\partial {C}_{{\rm{c}}}}$$

To assess the effects of chloroplast number and size on changes of photosynthetic limitation in each ecotypes, the relative limitations were linked to overall changes in *A*:

15 $$\frac{dA}{A}={L}_{s}+{L}_{m}+{L}_{b}=\frac{d{g}_{s}}{{g}_{s}}{l}_{s}+\frac{d{g}_{m}}{{g}_{m}}{l}_{m}+\frac{d{V}_{cmax}}{{V}_{cmax}}{l}_{b}$$

where *L*_s_, *L*_m_ and *L*_b_ are the reduction fractional limitation in *A* caused by reduction in stomatal conductance, mesophyll conductance and biochemistry, respectively. In the current study, the photosynthetic parameters in two wild type were defined as the references. The *g*_m_ values from Harley method and the *V*_cmax_ from A-Cc curves were used in calculations.

### Quantitative limitation analysis of mesophyll conductance

To quantify the main structural limitations of *g*_m_, an analogous analysis of Tosens *et al*.^32^ and Tomas *et al*.^26^ was applied. In the current study, the gas phase and structural components of *g*_m_ (*g*_i_) were estimated from Eqn 8–11. The gas-phase limitation of *g*_m_ (*l*_ias_) was calculated as:

16 $${l}_{{\rm{ias}}}=\frac{{g}_{{\rm{m}}}}{{g}_{{\rm{ias}}}}$$

The structural components limitation of the cellular phase conductances (*l*_i_) was estimated as:

17 $${l}_{{\rm{i}}}=\frac{{g}_{{\rm{m}}}}{{g}_{{\rm{t}}}\cdot {S}_{c}}$$

with *l*_i_ representing the limitation by the cell wall, the plasmalemma, cytosol, chloroplast envelope and stroma. The limitation imposed by each cellular component was scaled up with *S*_c_.

### Statistical analysis

One-way ANOVA analysis was used to test the differences in measured traits (in Tables) between estimated genotypes. All analyses were performed in R version 3.3.1 (https://cran.r-project.org).
